# Service Learning in the Nursing Bachelor Thesis: A Mixed-Methods Study

**DOI:** 10.3390/ijerph191912387

**Published:** 2022-09-29

**Authors:** Judith Roca, Silvia Gros Navés, Olga Canet-Velez, Jordi Torralbas-Ortega, Glòria Tort-Nasarre, Tijana Postic, Laura Martínez

**Affiliations:** 1 Department of Nursing and Physiotherapy, University of Lleida, 25003 Lleida, Spain; 2 Health Care Research Group (GRECS), Biomedical Research Institute of Lleida, 80 Alcalde Rovira Roure St., 25198 Lleida, Spain; 3 Faculty of Health Sciences Blanquerna, University Ramon Llull, 08022 Barcelona, Spain; 4 Global Health, Gender and Society Research Group (GHenderS), 326-332 Padilla St., 08025 Barcelona, Spain; 5 Nursing Care Research Group, Sant Pau Biomedical Research Institute (IIB SANT PAU), 08041 Barcelona, Spain; 6 CAP Calaf. SAP ANOIA, Gerència Territorial Catalunya Central, Institut Català de la Salut (ICS), 25600 Lleida, Spain; 7 AFIN, Research Group and Outreach Centre, Universitat Autònoma de Barcelona, 08193 Barcelona, Spain; 8 Igualada University Hospital, University of Lleida, 25003 Lleida, Spain

**Keywords:** bachelor thesis, evidence-based practice, nursing, service learning, student

## Abstract

The Final Degree Project (FDP) is a module that, although intended for the completion of a bachelor thesis (BT), consists of theoretical and clinical teaching. Therefore, introducing service learning (SL) can support student adjustments to the real-world professional role. This study plans to evaluate a teaching innovation project that combines BT and SL through Kirkpatrick’s four-level model (reaction, learning, behaviour and results). It takes the form of a convergent parallel mixed-methods design study. The participants were 15 final-year students obtaining a Bachelor of Nursing degree, 4 BT supervising mentors and 4 nurses. At the request of a hospital institution, in their BT, students completed a review of evidence-based nursing protocols. For data collection, the researchers used: an SL questionnaire, student narratives, mentor field diaries and nurse interviews. According to student opinion, the results showed high satisfaction rates (4.44 out of 5), the most developed skills were Independent Work and Information Management, but they signal the need to reinforce the research methodology skills. Finally, positive feedback from all participants is that using SL promotes both the opinion that the BT is useful and also promotes a collaboration between academic and clinical settings.

## 1. Introduction

University education should be aimed at providing skills that give students a critical assimilation of information that allows them to be more autonomous, independent and self-regulated. That is, schooling that really enables them to learn how to learn [1]. In the academic context, the subject of the Final Degree Project (FDP) offers a relevant learning context and therefore an opportunity for the student to demonstrate the skills developed during nursing training [2]. This is a module that, although intended for the completion of a bachelor thesis (BT), consists of theoretical and clinical teaching and assesses the intended learning outcomes over the course of the degree [3,4]. In most European Union countries, this module is a compulsory part of the final year, totalling between 6 and 12 credits under the European Credit Transfer and Accumulation System (ECTS), depending on the course (equivalent to 150 to 300 h of study) [2,5]. However, not all international higher education institutions require nursing students to write a BT and sit the subsequent viva examination [6]. The BT should be based in the professional context of each degree title [7] and promote student adjustment to and awareness of the real-world professional role [8]. Consequently, it allows students to demonstrate their understanding [9] and see that what they have learned can be transferred to nursing practice [10].

Alongside the professional nursing environment, a teaching methodology that this frame of the BT can foster is that of service learning (SL). SL allows experience-based learning in a practical environment, combining academic learning with community care [11]. Several studies have demonstrated the advantages of SL in various aspects of the field of nursing, such as: in cultural regards [12]; in care and social responsibility or self-care [13], as well as the fostering of reciprocal learning in the student–community dyad [14]. This strategy promotes trust and motivation [15], and consequently, by training in “reality”, learning becomes experiential and meaningful [16].

There have been no studies found in the literature review that combine the BT and SL, although some successes have been noted in other sources, with the results showing improvements in competency [17]. It can be highlighted that, being the greatest representation of the acquired professional competencies of a graduand, the BT is the ideal learning space to carry out SL [2,3]. The student can perform a genuine act of service using more consolidated knowledge and under less supervision [18] and can give this acquired knowledge back to the community and the profession in the form of service.

A combined experience of the BT and SL, such as that detailed in this teaching innovation project, can support the development of another basic element: evidence-based practice within the nursing discipline. This would help to face situations from an optimally informed perspective, improving decision-making, nursing practice and patient outcomes [19]. Students can support professionals in overcoming some of the implementation barriers associated with evidence-based practice within clinical settings, such as those regarding technology and methodology [20]. Moreover, the potential of this teaching innovation project is based in the contextualisation that is achieved with the BT and SL, wherein the student learns evidence-based practice in the most theoretical academic environment and puts it into practice upon moving to the clinical context [21]. It is in the later years that students show more preparation and readiness to develop their knowledge, skills and attitudes related to evidence-based practices [22]. 

Finally, it should be noted that the Kirkpatrick’s training evaluation model [23] will form the backbone of the evaluation for this learning method. The levels are: Level 1 (reaction), which facilitates the assessment of the key characteristics of the BT learning module; Level 2 (learning), which measures the skills and competencies acquired; Level 3 (behaviour), which evaluates if participants use acquired knowledge in context and Level 4 (results), which assesses the impact of training when moving to the workplace.

Therefore, the primary objective of this study is to evaluate a teaching innovation project that combines SL and BT through the Kirkpatrick’s four-level model (reaction, learning, behaviour and results). 

## 2. Methods

### 2.1. Study Design

This study used a convergent parallel mixed-methods design [24]. In this research approach, quantitative and qualitative data are collected and analysed at the same time. 

### 2.2. Description of the Teaching Innovation Project

During the planning of this teaching innovation project, a new type of BT was brought forward, which consisted of a revision of hospital protocol following evidence-based research criteria (see Supplementary Materials: Brief protocol development guidelines). In this way, the SL was incorporated with the BT. 

Students were free to choose the type of their BT and the protocol they would develop. The IUH previously supplied a list of potential nursing protocols for revision according to their established needs. 

Students chose their topic in October. During the writing process, they completed one tutorial session per month and met with expert nursing staff in the hospital to consult with them as needed. In May, each student handed in their BT, and in June, the live BT viva took place, as with all other students undertaking a BT module. Once the process was finalised, the completed project was delivered to the hospital institution for its final assessment. 

### 2.3. Study Context

The framework for this investigation is the Final Degree Project (FDP) module, in which a BT is written during the fourth, and last, year of the Bachelor of Nursing degree at the University of Lleida’s Faculty of Nursing and Physiotherapy. It is worth 9 ECTS credits (225 h). In Spain, the Bachelor of Nursing degree lasts 4 years at 240 ECTS (roughly 6000 h), and this module is compulsory. 

The Igualada University Hospital (IUH), the central hub of the Igualada campus, offered to act as the host service centre for the duration of this study. Students revised the nursing protocols at this hospital.

### 2.4. Study Participants

Participants were recruited through purposeful sampling of convenience specifically [25]. The participant eligibility criteria were as follows: participants must be students who will write a BT incorporating SL, they must participate voluntarily and they must be able to report on the various dimensions of the project. The clinical nurses were selected based on their expertise in each protocol specialty. The academic mentors formed the investigation group and were responsible for tutoring the students who had chosen this type of BT for their expertise in this SL methodology. In total, there were 23 participants: 15 nursing students, 4 mentors and 4 working nurses. All of the participants were women (23 out of 23). The mean age of the students was 21.78 years old (SD = 1.39); of the mentors, it was 50 years of age (SD = 4.5) and of the working nurses, the mean age was 41 years old (SD = 3.5). Regarding the mentors, 4 out of 6 had PhDs, and all of them had extensive teaching experience mentoring the BT (mean = 7.33 years, SD = 2.88). The working nurses were clinical professionals in a hospital institution, with a mean of 45 years’ work experience (SD = 15), and 6 out of 6 were educated to the Master’s or postgraduate level.

### 2.5. Data Collection

To assess this teaching innovation project, the following data collection plan was adhered to:

(1) Upon starting the module, students provided a narrative account of their expectations and again upon completion of the module to discuss whether they had met these expectations. At this final stage, they also completed a questionnaire about SL, created in Spanish and validated in an academic university context, the dimensions of which demonstrated adequate reliability (0.871–0.941) [26]. This questionnaire covered 6 dimensions and had 16 questions in total. However, to present the results of this study, only two of these dimensions were selected: general and soft skills (questions 7 and 8) and overall satisfaction (questions 13–16). The other questions referred directly to SL and were outside of the Kirkpatrick’s training evaluation model [23]. In addition, the questionnaire contained 4 open questions to garner student opinions of their experience completing the BT, such as what completing this work meant to them, the experiment, meeting expectations and areas for improvement.
(2) During the process, the mentors measured the students’ progress through use of a field diary as a means of observing the experiment.
(3) Clinical nurses were interviewed by researchers. A semi-structured interview was carried out covering the following areas: assessment of the innovation, utility and strategies to improve the student–mentor–nurse working relationship.

Table 1 lists Kirkpatrick’s four levels alongside the associated data collection instruments and the time of collection. In total, 55 documents were analysed. Data collection ended in June 2022.

### 2.6. Data Analysis

For the quantitative analysis, the researchers used measures of central tendency (means and SDs) and percentages using IBM’s SPSS program (SPSS Statistics V24.0, IBM Corp., Armonk, NY, USA). The qualitative analysis was performed using the content analysis technique [27] supported by the Atlas-ti V7 program. The units of analysis were selected, and for clarity and conciseness, the categories and themes were created afterwards.

To ensure that the credibility, dependability and transferability criteria were met [27,28,29], a series of actions took place during the qualitative analysis of the data: (1) the sampling of participants allowed for a diverse range of information and a response to the planning of the research; (2) the context and participants were reported on and (3) the research team reviewed the units of meaning and the abstraction, condensation and category creation processes.

### 2.7. Ethical Considerations

This study was assessed positively by the Research Commission at the Faculty of Nursing and Physiotherapy at the University of Lleida and was granted permission to move forward by the IUH. Participants were asked for consent. Data confidentiality and anonymity were guaranteed throughout the entire study by assigning each document and participant an alphanumeric code.

## 3. Findings

The results were presented according to the four levels proposed by Kirkpatrick’s four-level model (reaction, learning, behaviour and results). Level 1 permits an evaluation of the key learning principles before and after completion of the BT according to student opinion. Table 2 details the results of the student narrative analysis (beginning and end). 

In Level 2, learning is measured through acquired knowledge and skills and overall student satisfaction. The students rated both the skills they attained through the BT and their degree of involvement as 4.44 out of 5 (SD = 0.73) (Table 3).

Table 4 details the most developed soft skills according to the students. Independent work and Information management are the two highest rated skills at 4.44.

Level 3 assesses whether students have applied their acquired knowledge or not and its utility in context. Table 5 details the results of the student narrative analysis in relation to the connected skills.

Finally, Level 4 involves assessing the impact of training. This section is rated by mentors and nurses. Table 6 presents the results of the field diaries of the teaching staff and of the nurse interviews.

## 4. Discussion

This mixed-methods study presents its results by means of an evaluation of a teaching innovation project for a Bachelor of Nursing degree, which incorporates a SL teaching strategy in the BT writing process. For this, the Kirkpatrick’s four-level model (reaction, learning, behaviour and results) was used as a frame of reference. This model allows us to integrate different participants (students, mentors and working nurses) and different stages of learning into the analysis. It also simplifies the assessment of training programs and services [30,31]. 

Regarding Level 1 (reaction), students highlight the utility of the task, i.e., a revision of an evidence-based nursing protocol, by its relation to clinical practice and its potential for later use in healthcare. Some studies discuss BT typology [2,32], but the BT does not feature this. In this respect, as well as in the use of SL, is this project’s innovative nature. The use of SL reinforces the ongoing collaborative relationship between theory and practice [15]. Furthermore, according to Anderson, Boyd, Ariemma Marin and McNamara [33], SL bridges the gap between academic results and the inherent value of performed service. Another aspect that students highlight is the learning that has taken place, reinforcing the stance of Jefferies et al. [6], who claimed that writing a BT is an exercise in academic literacy. Moreover, in dealing with an evidence-based protocol, the SL strengthens two key aspects of the BT: professional development and the integration of evidence-based practice [34]. Offering different types of BT allows for a richer and more multifaceted approach in undergraduate nursing education [21]. 

Learning through acquired skills and knowledge, as well as student satisfaction, are measured in Level 2. Students highlight that their most developed skills are Independent Work, thereby enabling them to manage their own learning [35]. Other skills that are developed according to the students’ perceptions are related to those described by other authors, such as the ability to analyse and synthesise, search for and manage information, autonomous work, communication, critical reasoning and ethical commitment [36]. These results are consistent with those of other studies [2,8,10,37]. Furthermore, in Henttonen et al. [38] about expectations when writing a thesis, the nursing students hoped to obtain valuable knowledge for professional practice. In our study, they attained this knowledge, reflected by their high satisfaction rate, although the writing process generated anxiety and difficulty due to a specific lack of training [39].

Level 3 explores skills and their utility in context. Students feel that the BT allows them to apply a great deal of the knowledge and resources they have gathered during their degree, and therefore, it complies with the current Spanish legislation [40]. However, they note a lack of training in research and methodology skills. The process of writing a BT helps introduce students to scientific activities [41] and helps to develop a positive attitude towards research [42] by creating opportunities to do so [43]. Developing research and evidence-based practice skills during their nursing training helps students value the importance of research and its applications in clinical settings, resulting in positive patient outcomes [44,45], or for students who wish to work in research [46], it acts as an introduction to this option [38].

Finally, Level 4 measures the impact of training according to BT mentors and expert nurses assessing the performed work. Just like students, both working nurses and mentors highlight the utility of the project and the potential for collaboration. The BT offers the opportunity to develop research skills in a guided academic environment [47] and, in our case, also in a clinical setting, where nurses take part in the process alongside students. Ryan [43] emphasises the importance of ensuring nurses support students in their research. Furthermore, this type of BT offers students the possibility to take initiative and introduce small improvements to clinical nursing practices [46], motivates everyone involved in the process and allows for discipline-relevant topics to be discussed [48]. Finally, it is essential that any educational protocol promotes constructive interactions between participants (tutors and students) to achieve the learning outcomes proposed in a BT [49].

## 5. Conclusions

The results of this teaching innovation project combining the BT and SL demonstrates that all participants (students, mentors and working nurses) find it useful and that there are collaborative opportunities between academic and clinical practice and the potential for professional career advancement, as well as the possibility of improved student competency and future specialisation in the field of research and evidence-based practice. That said, however, the research methodology skills should be further developed within nursing training.

## Figures and Tables

**Table 1:** Summary of the instruments, participants and data collection following the Kirkpatrick model.

	Instrument	Participants	Data Collection	Data Analysis
Level 1: Reaction	Narrative about expectations before and after	Students	Before and at the end of the experiment	Qualitative (content analysis)
Level 2: Learning	Questionnaire about SL (competency and satisfaction)	Students	At the end of the experiment	Quantitative (means and standard deviations)
Level 3: Behaviour	Open questions from the questionnaire about SL	Students	At the end of the experiment	Qualitative (content analysis)
Level 4: Results	Semi-structured interview	Nurses	At the end of the experiment	Qualitative (content analysis)
	Field diary	Mentors	Continuously throughout	

**Table 2:** Qualitative results matrix, Level 1 (participant student: PS).

Area of Explored Content	Categories	Definitions	Units of Meaning
Characteristics of the BT	Utility	Value placed in the BT	*“…I feel like my work is useful if it can be applied to clinical practice and not just be something I remember or something I can show to the tribunal.”* (PS1) *“…the BT is innovative for the whole nursing field; it is well recognised and its usefulness goes beyond the personal level.”* (PS8)
	Complexity	Perception of difficulties and overcoming them	*“…completing this task requires a lot of determination and thorough research for relevant, high-quality information.”* (PS4) *“…it’s a very current topic and the fact you create it from scratch implies more dedication and time spent.”* (PS6)
BT as a process	A learning process	The end process in which the BT is viewed as a source of training and competence development	*“This research has positively impacted my learning as a future nurse (learning new skills in a topic, carrying out protocols, performing techniques step-by-step, finding up-to-date scientific information with the most possible evidence, etc.)…”* (PS5) *“…it’s a task where you have to be on top of so many aspects: presentation, reliable articles, databases, up-to-date information… It’s the last academic step before becoming a nurse.”* (PS3)
	A professionalising process that goes hand-in-hand with clinical practice	A process of continuity between theory and clinical practice and the shaping of the profession	*“…as students and future nurses, we have to empower our situation and make nursing more public and more recognised.”* (PS7) *“…the creation of a nursing protocol has also motivated me to carry it out, as it could be used within real healthcare practice.”* (PS8)

**Table 3:** Quantitative results: satisfaction rates, Level 2 (students).

(1 = Very Unsatisfied; 2 = Unsatisfied; 3 = Neutral; 4 = Satisfied; 5 = Very Satisfied)	M *	SD *
Project planning	4.00	1.12
Institution involvement	3.33	1.22
Student involvement	4.44	0.73
Learning outcomes achieved	4.44	0.73
Relationship between theory and practice	4.33	0.87
Assessment carried out	3.33	1.12
Learning activities carried out	3.89	1.05
Resources for performing activities	3.44	1.24
Activity schedules	3.33	1.50
Participants I do activities with	3.89	1.17
Coordination between mentors and institution	4.00	1.41
Mentor follow-up	4.11	1.45
Service carried out	4.22	0.97

Note: * (M): median and (SD): standard deviation.

**Table 4:** Quantitative results: soft skills assessment, Level 2 (students).

(1 = Very Unsatisfied; 2 = Unsatisfied; 3 = Neutral; 4 = Satisfied; 5 = Very Satisfied)	M *	SD *
Analysis and summary skills	4.67	0.50
Knowledge and understanding of ideas or concepts	4.56	0.73
Organisation and planning	4.56	0.73
Information management	4.44	0.72
Independent work	4.44	0.88
Oral and written communication	4.33	0.71
Upholding ethical responsibility	4.11	1.05
Caring about quality and improvement	4.11	1.05
Critical thinking	3.89	1.17
Designing and managing projects	3.78	1.09
Decision making	3.67	1.00
Adapting to new situations	3.67	1.12
Being creative and innovative	3.67	1.22
Assessing sustainability of proposals and performance	3.67	1.00
Showing initiative and a forward-thinking attitude	3.56	1.13
Problem solving	3.33	1.22
Assessing social and environmental impact of performance	3.22	1.56
ICT skills	3.00	1.73
Leadership	3.00	1.41
Foreign language skills	2.75	1.49
Recognising diversity and multiculturalism	2.67	1.87
Working in a team	2.56	2.19
Expressing feelings	2.44	1.51
Negotiation	2.00	2.12

Note: * (M): median and (SD): standard deviation.

**Table 5:** Qualitative results matrix, Level 3 (participant student: PS).

Area of Explored Content	Categories	Definitions	Units of Meaning
Applicability of skills in context	Acquired skills	Application of skills gained throughout training	*“…the BT has allowed me to develop many skills related to evidence-based practice, biostatistics, pathophysiology, anatomy, physiology, and nursing care.”* (PS11) *“…applying general skills such as summarisation, prioritising information, knowing how to reference, good use of language, among others.”* (PS13)
	Useful skills	Satisfaction with certain skills considered to be convenient or useful in completing the BT	*“…the fact we’ve been able to do so many written tasks throughout the degree has also improved how we summarise and present our ideas.”* (PS10) *“Not only are acquired skills important, as they’re the foundation of our performance as nursing professionals, but also how we apply these concepts and the skills we learned during this time.”* (PS10)
	Skills for further development	Incorporating new skills to improve the BT process	*“…more training in research skills would be needed and also how to do certain tasks, like protocols.”* (PS11) *“…we’re missing methodology. I think we have very superficial knowledge to be writing a BT.”* (PS12)

**Table 6:** Qualitative results matrix, Level 4 (participant nurse: PN and participant mentor: PM).

Area of Explored Content	Categories	Definitions	Units of Meaning
BT as a final curricular process	Learning	Value as a means to assess student competence acquired throughout education	*“I think that apart from bringing together the different learning outcomes of the degree in a more theoretical and academic way, it also allows students to branch into research skills in a practical manner.”* (PM2) *“It’s clear that the BT functions as a final assessment method in the nursing degree.”* (PM2)
	Utility	Value of the BT when incorporated into clinical practice and for party cooperation (mentors and nurses)	*“… [the BT has] value not only for its innovative nature, but also for the learning potential and transferable skills to clinical practice.”* (PN6) *“It lets academics and clinicians work together in a team [through] shared tutorial sessions.”* (PN2)
Facilitators and barriers in incorporating SL and the BT	Facilitators	Aspects that have helped develop this category of BT	*“It increases satisfaction rates among students.”* (PM2) *“…student motivation has been outstanding.”* (PM1)
	Barriers	Aspects that have created difficulties	*“More work for mentors in terms of hours, despite the economic benefits…”* (PM1) *“Coordination between different parties has to improve to achieve a higher quality product.”* (PN5)

## Data Availability

Data are contained within the article.

## Supplementary Materials

The following supporting information can be downloaded at https://www.mdpi.com/article/10.3390/ijerph191912387/s1: Protocol development guidelines.
