# Supplementary material for: Service Learning in the Nursing Bachelor Thesis: A Mixed-Methods Study
Source: Int J Environ Res Public Health. 2022 Sep 29;19(19):12387. doi: 10.3390/ijerph191912387 (PMC9566517; doi:10.3390/ijerph191912387)
Supplement: Supplementary file 1 [file ijerph-19-12387-s001.zip › ijerph-1903607-supplementary.pdf]

International Journal of *Environmental Research and Public Health*

MDPI

Supplementary Material

Protocol development guidelines

Based on a Protocol or Procedural Review, the structure of the BT will be as follows:

Title page

Acknowledgements or Dedications (optional)

Table of Contents

Includes the study content index, the figures, graphs and tables index, and the index or list of abbreviations.

Abstract

Written in Catalan, Spanish and English. Maximum wordcount of 300 words.

There is a maximum of 5 keywords that must, whenever possible, correspond to MeSH or DeCS descriptors.

1. Introduction

1.1 Rationale

2. Narrative review

2.1. Theoretical framework

- Description of topic to address
- Antecedents and current state of the topic. Critical review of evidence.
- Nursing theory: appropriate framework for protocol development.
- NANDA diagnoses, NIC interventions and related NOC outcomes.

3. BT Objectives

4. Methods

4.1 Research methodology

4.2 Protocol creation or revision methodology.

4.3 Establishing phases and timeline. Describe each phase. Group work. Estimate of costs.

5. Results

5.1 Protocol

- Date created
- Authors
- Reviewers
- Conflict of interest
- Introduction
- Protocol Name
- Terms and definitions/glossary
- Definition
- Protocol objectives
- Indications
- Healthcare field or procedure
- Target population
- Activity to perform / Plan of action / Procedure. Level of evidence of interventions/proposed activities
- Algorithms for action
- Human resources and materials

- Record-keeping systems
- Assessment and Indications

Name of indicator	
Owner of indicator:	
Type of indicator:	
Objective / rationale:	
Formula:	
Explanation of terms:	
Population:	
Responsible for collection:	
Collection frequency:	
Observations / Comments:	

- Protocol bibliography
- Protocol appendices

5.2. Implementation plan

6. Participant reflective report of learning

7. Conclusions

Limitations.

8. References

9. Appendices
